# Synergistic Integration of Liposomes with Emerging Technologies for Food Applications

**DOI:** 10.3390/molecules31071160

**Published:** 2026-03-31

**Authors:** Miguel A. Varas Condori, Aarón Ibáñez Bendezú, Jaime Romero, Alejandro Villasante, Rafael Opazo, Jimena Cordero-Machuca, Cristina Muñoz-Shugulí, Cristian Patiño Vidal, Ricardo Andrade-Pizarro, Johana López-Polo

**Affiliations:** 1 Laboratorio de Biotecnología de los Alimentos, Instituto de Nutrición y Tecnología de los Alimentos, Universidad de Chile, Santiago 7830490, Chile; 2 Dirección de Tecnologías de Información, Universidad Central de Chile, Santiago 8370178, Chile; 3 Facultad de Medicina Veterinaria y Agronomía, Universidad de Las Américas, Santiago 7500000, Chile; 4 Department of Science and Pharmaceutical Technology, Faculty of Chemical and Pharmaceutical Science, Universidad de Chile, Santiago 8380494, Chile; 5 Escuela Superior Politécnica de Chimborazo (ESPOCH), Facultad de Ciencias, Riobamba EC060155, Ecuador; 6 Safety and Resources Valorization Research Group (INVAGRO), Faculty of Engineering, Universidad Nacional de Chimborazo (UNACH), Riobamba EC060107, Ecuador; 7 Group for Research and Innovation in Food Packaging, Riobamba EC060107, Ecuador; 8 Departamento de Ingeniería de Alimentos, Facultad de Ingeniería, Universidad de Córdoba, Berástegui 232527, Colombia

**Keywords:** liposome, edible coating, edible film, electrospinning, cyclodextrin

## Abstract

Food by-products have gained importance as valuable sources of bioactive compounds and structural lipids, with potential applications in food packaging. These residues, such as fruit peels, seeds, and fish skin, contain polymers and natural compounds like polyphenols, carotenoids, tocopherols, and phospholipids, which possess antioxidant and antimicrobial properties highly relevant for food preservation. However, the direct incorporation of these compounds is limited by their sensitivity to environmental factors such as light, oxygen, and pH. Liposomal encapsulation has emerged as a promising strategy to overcome these challenges, providing protection, controlled release, and increased bioavailability of both hydrophilic and lipophilic bioactives. The formulation of liposomes using lipids recovered from food industry by-products introduces an additional sustainability component, in line with the principles of the circular economy. Combining liposomes with other advanced preservation technologies, such as edible coatings and films, electrospinning fibers, and cyclodextrin inclusion complexes, is a promising alternative for extending the shelf-life and safety of food products, as well as for the development of functional foods. This review discusses the latest advances in liposome formulations with food by-products and their combination with other technologies to enhance their effectiveness in food preservation.

## 1. Introduction

Traditional food packaging materials such as plastic, aluminum, and cardboard, although effective in protecting food, present limitations in terms of recyclability, biodegradability, and long-term environmental impact. Consequently, the search for bio-based, sustainable, and environmentally friendly alternatives has intensified in recent years, focusing on materials derived from renewable sources that not only protect food but also help reduce by-products and the environmental footprint [1,2]. An example of this is the growing interest in the development of biodegradable films and coatings made from natural biopolymers. These matrices are not only biodegradable and compostable but can also act as carriers of active compounds, such as antioxidants and antimicrobials, which extend the shelf-life of food by slowing oxidation and microbial spoilage processes [3,4].

On the other hand, the valorization of food waste and by-products has emerged as a priority area within the circular economy. Large volumes of agro-industrial by-products, such as fruit peels, seeds, pomace, crustacean shells, and cereal residues, contain high-value compounds that, instead of being discarded, can be recovered and reused to develop new food products or functional materials [5,6]. These by-products are natural sources of proteins, biopolymers, and bioactive compounds, including polyphenols, flavonoids, carotenoids, tocopherols, proteins, and structural lipids [7,8]. Such molecules possess antioxidant, antimicrobial, and anti-inflammatory properties, making them ideal candidates for incorporation into active packaging systems or functional food formulations aimed at enhancing consumers’ health [9].

However, the direct use of these bioactive compounds presents technological limitations due to their instability when exposed to environmental factors, such as light, oxygen, humidity, and pH, resulting in degradation and, consequently, the loss of functional activity [3]. The chemical stability of polyphenols and other phytochemicals is closely linked to their molecular structure, particularly the presence of hydroxyl groups, conjugated systems, and glycosylation patterns, which determine their reactivity toward environmental stressors [10]. Light exposure accelerates degradation through photoinduced reactions, including isomerization and oxidation, often mediated by photosensitizers such as riboflavin via Type I and Type II mechanisms, or through direct light absorption [10,11]. Oxygen further promotes instability through autooxidation, leading to peroxide formation and subsequent oxidative degradation, while pH variations induce structural transformations (especially in anthocyanins) where acidic conditions favor stable flavylium forms and alkaline environments accelerate degradation [12]. Elevated temperatures also contribute to instability by inducing reactions such as epimerization during processing and storage [10].

In this regard, encapsulation emerges as an effective tool for protecting sensitive compounds and ensuring their functionality during food processing and storage [13]. Among the various encapsulation techniques available, liposomes stand out for their versatility, biocompatibility, and ability to encapsulate both hydrophilic and lipophilic compounds [14]. Liposomes are spherical vesicles formed by one or more lipid bilayers, mainly composed of phospholipids and cholesterol. In the food industry, these systems have been successfully used to encapsulate bioactive compounds, such as polyphenols, essential oils, plant extracts, vitamins, and natural pigments [15]. Their structure allows for the protection of encapsulated compounds from adverse conditions, improves bioavailability, and enables controlled release, making them highly advantageous for functional foods and active packaging applications [14,16].

The possibility of formulating liposomes using lipids extracted from food by-products adds an additional dimension of resource efficiency to the encapsulation process. By-products such as fish skin and liver, shrimp shells, or egg yolk residues contain natural phospholipids that can serve as raw materials for liposome formation [17,18]. This strategy not only reduces production costs but also promotes the recovery and full valorization of agro-food residues, supporting the circular economy and reducing the environmental impact associated with the industry. Furthermore, the incorporation of liposomes into emerging technologies, such as edible coatings and films, electrospinning, and cyclodextrin inclusion, represents a promising strategy for enhancing food preservation. These combined systems facilitate the controlled release of active compounds and improve their distribution throughout the packaging matrix or on the food surface, providing additional protection against oxidation and microbial growth [3,4].

Although liposomes, edible coatings and films, electrospinning, and cyclodextrin inclusion complexes have been extensively reviewed as individual technologies, there is still limited integrative analysis addressing their combined application in food systems, particularly when liposomes are formulated using lipids recovered from food and agro-industrial by-products. Therefore, the objective of this article is to review, update and discuss the applications of liposomes in food preservation, analyzing the use of food by-products in their formulation and their combination with complementary technologies, such as edible coatings and films, electrospinning, and cyclodextrins. Finally, emphasis is given to highlight how these synergies improve the functionality of liposomes, increasing the stability of bioactive compounds and extending the shelf-life of food products.

## 2. Liposomes

Liposomes can encapsulate hydrophilic, lipophilic, or amphiphilic compounds within lipid bilayer vesicles, enabling diverse food applications [19]. Liposomes offer multiple benefits and are utilized in a wide range of products within the food and pharmaceutical industries. Their structure consists of lipid-based vesicles, typically composed of phospholipids and cholesterol, which can vary in size and number of layers [20]. The main advantage of employing this type of technology lies in its capacity to encapsulate and protect bioactive molecules, which is particularly beneficial for compounds, such as polyphenols, that are sensitive to light, temperature, gases, and other environmental stressors [21].

Liposomes are mainly composed of phospholipids such as phosphatidylcholine (lecithin), phosphatidylethanolamine, phosphatidylserine, and phosphatidylglycerol [22]. In some cases, liposome modulators such as cholesterol, bile salts, nonionic surfactants, and chitosan are added to enhance the structural stability and rigidity of liposomes [19]. The properties of liposomes depend on the composition of phospholipids. Unsaturated phosphatidylcholine species derived from natural sources (egg or soy) yield more permeable and less stable bilayers, whereas the use of saturated phospholipids with long acyl chains, such as dipalmitoylphosphatidylcholine, results in more rigid and impermeable bilayer structures [23].

**Figure 1:**
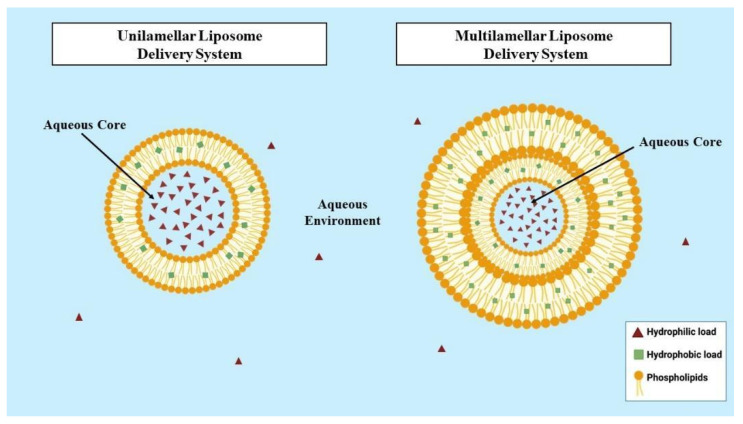
Types of liposomes and their charge based on lamellarity: Unilamellar Liposome Delivery System (**left**) and Multilamellar Liposome Delivery System (**right**). Created with BioRender.com.

Liposomes are primarily classified according to their lamellarity (number of bilayers) and size [24]. Based on their lamellarity, liposomes are classified as unilamellar vesicles (containing a single bilayer), oligolamellar vesicles (with two to five bilayers), and multilamellar vesicles (comprising more than five bilayers). In terms of size, unilamellar vesicles can be small (20–100 nm), large (>100 nm), or giant (>1 mm), whereas multilamellar vesicles exceed 0.5 mm, and oligolamellar vesicles range from 0.1 to 1 mm. The number of bilayers and the size of the liposomes directly influence the amount of drug or active compound that can be encapsulated [25]. Depending on the encapsulation objective, the most suitable type of liposome is selected. Large unilamellar vesicles, due to their higher internal volume, are ideal for encapsulating hydrophilic molecules, while multilamellar vesicles, with multiple hydrophobic regions in their bilayers, are particularly effective for incorporating lipophilic molecules (Figure 1) [24].

### 2.1. Liposome Production Techniques

Regarding liposome production, it is important to understand that this process is divided into two main stages: the first corresponds to liposome formation itself, and the second involves post-production techniques [26]. Liposome production methods include thin-film dehydration–hydration [27], solvent injection methods [28,29], microfluidic technologies [30], the detergent removal (depletion) method [31], and heating techniques [32]. These methodologies are comprehensively reviewed in the work by Lombardo and Kiselev [33].

Methods for obtaining liposomes generally include four stages: drying a mixture of lipids in an organic solvent, dispersing the lipids in aqueous media, separating and purifying the formed liposomes, and analyzing the final product [23]. The thin-film method is the most widely used technique for liposome preparation. It involves forming a lipid film in a rotary evaporator, which is then hydrated with water or a buffer solution preheated above the lipid phase transition temperature. This process, combined with vigorous agitation, generates multilamellar liposomes. Lipophilic compounds are mixed with the lipids, while hydrophilic compounds are added to the aqueous phase. From the multilamellar liposomes, small unilamellar liposomes can be formed through cell disruption using high-intensity ultrasound [34]. Several conventional techniques exist for liposome preparation, including reverse-phase evaporation, injection, hydration, microfluidics, and extrusion. However, these methods often present disadvantages that limit their application at an industrial level, such as complex procedures, low encapsulation efficiencies, the risk of denaturing active compounds under adverse conditions, and the use of organic solvents that must be eliminated for food applications [23]. In this regard, homogenization techniques using ultrasound and heating offer good encapsulation efficiencies, use only food-grade solvents, and employ simple, easily scalable procedures. However, energy costs at an industrial level still need to be evaluated.

### 2.2. Valorization of Food By-Products in Liposome Formulation

In recent years, the use of agri-food by-products and waste as sources of lipid and bioactive compounds for liposome formulation has attracted growing scientific and technological interest. Several studies have demonstrated that residues such as fish skins and viscera, shrimp shells, and by-products from the vegetable oil industry contain phospholipids, other structural lipids, and phenolic compounds that can be extracted and employed in the preparation of liposomes [35,36]. Table 1 shows works where different by-products of the food industry have been valued for the production of liposomes with different applications. This strategy not only reduces the use of high-cost or synthetic raw materials but also promotes a more sustainable approach aligned with the principles of the circular economy [37]. Furthermore, the technical feasibility of producing food-grade liposomes such as vesicles loaded with carotenoids obtained from soy lecithin has been demonstrated, highlighting the potential of combining lipids derived from by-products with liposomeapplicable in the food sector [38].

Additionally, agro-industrial residues are also an important source of bioactive compounds, such as polyphenols, carotenoids, flavonoids, tocopherols, and peptides, that possess antioxidant and antimicrobial properties of great interest to the food industry [39]. The encapsulation of these compounds in liposomes prepared from natural materials offers an effective solution to improve their stability against environmental factors, enhance their bioavailability, and enable controlled release in food matrices. This dual valorization of both structural lipids and bioactive compounds present in by-products represents a sustainable and innovative alternative for the development of functional ingredients and active packaging systems [40]. In this sense, liposomes stand out as a versatile technological tool that integrates environmental sustainability with technological functionality, supporting the transition toward a more efficient and environmentally responsible food industry.

However, a critical distinction must be made between raw material sustainability and process-level sustainability to avoid overgeneralizing the green nature of these systems. The use of biopolymers and liposomes in food applications relies primarily on the valorization of agro-industrial by-products as sustainable sources of polymers, structural lipids, and bioactive compounds, rather than on the entire manufacturing process [6] that often involves significant environmental trade-offs. For instance, conventional laboratory-scale methods for polymer and lipid extraction and liposome preparation remain heavily dependent on organic solvents and energy-intensive steps (rotary evaporation, sonication, etc.) for efficiency and reproducibility; these protocols are not classified as green technologies [41]. In this context, the ecological advantage for liposomes highlighted in this review lies in strategically replacing synthetic or high-cost compounds with waste-derived materials, thereby promoting waste reduction and a circular economy [42]. Consequently the term “sustainable” in much of the current literature predominantly refers to the sourcing of materials rather than the fabrication process. To address this gap, current research is increasingly focused on liposome production strategies that utilize reduced, solvent-free, or food-grade solvents to improve environmental compatibility and industrial scalability. These include supercritical CO_2_ extraction [43], ethanol-based systems using food-grade solvents suitable for large-scale production [44] and high-pressure homogenization [45], or methods like Jalilian et al.’s [46], which eliminate the use of toxic solvents and reduce energy requirements, thereby aligning the entire production chain with environmental compatibility.

**Table 1:** Valorization of food by-products in liposome formulation.

By-product	Active Compounds	Liposome Lipid	Liposome Preparation Method	Reference
Grape Pomace	Phenolic compounds	Brain lipids, biotin–PEG-DSPE	Thin-film evaporation method followed by extrusion	[47]
*Graciano* Grape Pomace	Phenolic compounds (anthocyanins, flavan-3-ols, and flavanols)	Phospholipid lipoid S 75 (S75)	Lipid dispersion with ultrasonic sonication	[48]
Merlot Grape Seed	Phenolic compounds (flavan-3-ols and proanthocyanidins)	Lipoid S100^®^ and cholesterol	Reverse-phase evaporation method with ultrasound and extrusion	[49]
Grape Seed Powder	Tannins	Phosphatidylcholine	Heating/homogenization method with size reduction by ultrasound cycles	[50]
Grape Pomace and Sea Buckthorn	Polyphenols and carotenoids	Egg yolk phospholipids	Heating method with sonication	[51]
Grape Seed	Procyanidins and α-tocopherol (coencapsulated)	Soybean lecithin	Modified ethanol injection method with sonication	[52]
Cannonau Grape Pomace	Polyphenol	Phospholipid S75	Direct dispersion of solid components in the aqueous phase, followed by sonication and purification by dialysis	[53]
Grape Seed	Condensed Tannins	L-α-Lecithin soybean (Phosphatidylcholine purity ≥ 97%)	Heating/homogenization process with size reduction by ultrasound cycles	[54]
Grape Pomace	Phenolic compounds	Soy lecithin granules	Dispersion of lecithin in buffer and probe sonication	[55]
Spent Coffee Grounds	Phenolic compounds	L-α-phosphatidylcholine	Supercritical-assisted liposome formation	[56]
Red Onion Peel	Anthocyanins	Soybean phosphatidylcholine	Thin-layer hydration method followed by sonication	[57]
Olive Mill Waste	Phenolic compounds	Phosphatidylcholine and cholesterol	Thin-film hydration method followed by extrusion	[36]
Olive Leaves and Orange Peels	Polyphenols (oleuropein, hydroxytyrosol derivatives, flavonoids, phenolic acids)	1,2-dioleoyl-sn-glycero-3-phosphocholine and cholesterol	Thin-layer evaporation method followed by sonication	[58]
Pineapple By-products (peels and pomace)	Phenolic compounds	Phospholipid P90G	Thin-film and rehydration method followed by sonication	[59]
Olive Leaves and Orange Peels	Polyphenols (oleuropein, hydroxytyrosol derivatives, flavonoids, phenolic acids)	1,2-dioleoyl-sn-glycero-3-phosphocholine and cholesterol	Thin-layer evaporation method followed by sonication	[60]
Green Tea Waste	Catechins	Soy lecithin (Beakin^®^ LV1 Lecithin)	High-speed homogenization followed by coating with chitosan	[61]
Wild Thyme Tea Processing Residues	Phenolic compounds	Phospholipon 90 G, Cholesterol and β-sitosterol	Proliposome method	[62]
*Araucaria angustifolia* Bract	Phenolic compounds	Phosphatidylcholine, cholesterol, and stearylamine	Lipid film hydration method, followed by sonication	[63]
Green Tiger Shrimp Waste	Bioactive peptides	Lecithin and cholesterol	Thin-layer hydration method followed by sonication	[64]
Shrimp Waste (head, shell, and tail)	Carotenoids from shrimp waste and fish oil	Lecithin	Homogenization (Ultraturrrax) followed by sonication	[65]

Table 1 shows that residues derived from the wine industry, such as grape pomace and grape seeds, represent the most extensively explored substrates for the extraction of phenolic compounds, anthocyanins, and condensed tannins [47,48,49,50,51,52,53,54,55]. This trend extends to other plant-based residues, including spent coffee grounds [56], red onion skins [57], olive leaves and olive mill waste [36,58], fruit processing residues [58,59,60], tea waste [61,62], and *Araucaria angustifolia* bracts [63], where the stabilization of secondary metabolites is pursued through the use of phospholipids such as phosphatidylcholine and soy lecithin. In addition to plant sources, the recovery of functional lipids and peptides from crustacean by-products, such as shrimp waste, has also been reported, involving the incorporation of carotenoids, α-tocopherol, and bioactive peptides into liposomal matrices [35,64,65]. Furthermore, Table 1 highlights that the thin-film hydration method (or thin-layer evaporation), followed by sonication or extrusion, predominates as the standard technique to ensure particle size reduction and vesicle homogeneity in liposome [47,48].

Nevertheless, the pursuit of higher encapsulation efficiency and improved scalability has driven the implementation of alternative methodologies, such as supercritical fluid-assisted formation [56], reverse-phase evaporation [49], and ethanol injection [60]. Another relevant aspect is that, in the formulation of these liposomal systems, the addition of components such as cholesterol or polymeric coatings with chitosan has been evaluated, as these may affect membrane rigidity and the controlled release of active compounds [49,58,61,64]. Despite the advances reported in these studies, variability in the purity of the lipids used—ranging from purified fractions to commercial lecithins—and the need for controlled thermal processes suggest that the standardization of synthesis protocols remains the main challenge for transferring these systems from laboratory scale to industrial production [35,49,61].

### 2.3. Liposomes and Their Application in the Food Industry

Liposomes loaded with bioactive compounds (leaf and algae extracts, essential oils, and oleoresins) possessing antioxidant and antimicrobial properties have been widely investigated for the preservation of different food systems, particularly highly perishable products such as marine and dairy products (Table 2). These bioactives can be efficiently incorporated into liposomal structures, improving their stability, distribution, and controlled release within food matrices [2,66].

Among marine products, fish filets and oysters have been studied due to their high nutritional value and susceptibility to lipid and protein oxidation. In tilapia filets, liposomes loaded with 1% ethanolic extract of *Piper betle* L. leaves effectively preserved the product over 12 days of storage at 4 °C, significantly reducing the proliferation of *Staphylococcus aureus*, *Escherichia coli*, *Pseudomonas aeruginosa*, and *Shigella sonnei* compared to a control without liposome addition [67]. Likewise, oysters, which are rich in high-quality proteins, polyunsaturated fatty acids, and other micronutrients, are highly susceptible to oxidative deterioration. Cheng et al. [2] utilized liposomes loaded with rosemary oleoresin to uniformly coat dried oysters by immersion for 2 min in a liposomal solution (0.5 mg/mL) [2]. This coating effectively delayed lipid and protein oxidation during 28 days of storage, as evidenced by lower peroxide and TBAR values (lipid oxidation indicators) and reduced carbonyl content (protein oxidation indicator) compared to dried oysters coated with liposomes lacking rosemary oleoresin [2].

In dairy products, liposomes have been explored as effective carriers for bioactive enrichment in cheese. Zanganeh et al. [66] reported that spirulina extract-loaded liposomes significantly reduced the growth of *Staphylococcus aureus* and *Listeria monocytogenes* in white cheese during storage at 4 and 25 °C. Gil et al. [68] incorporated *Thymus capitatus* L. essential oil into sheep cheese using soy lecithin-based liposomes (Lecinova^®^ and Lipoid S75). To reduce particle size and enhance stability, the liposomal dispersions were subjected to sonication, and homogeneous distribution within the cheese matrix was achieved by injecting six aliquots of 10 mL of essential oil-loaded liposomes into different regions of each cheese [68]. As a result, the enriched cheeses containing carvacrol and thymol maintained stable antioxidant capacity throughout 60 days of storage [68]. In fresh cheeses, Siyar et al. [69] incorporated saffron-extract-loaded nanoliposomes into ricotta cheese, achieving high physical stability (91.85–98.54%) and particle sizes of 155.9–208.1 nm using 0.5–4% soy lecithin. Liposome addition did not significantly affect pH, while yellowness (b*) increased with saffron extract concentration due to its carotenoid content, and hardness and chewiness increased at a saffron extract concentration of 0.125% compared to the control cheese [69].

In yogurt systems, liposomes have been evaluated as carriers for vitamins and plant bioactives under refrigerated storage and simulated gastrointestinal digestion. Ferreira et al. [70] incorporated lyophilized liposomes co-encapsulating vitamins D_3_ and B_12_ into yogurt without adversely affecting physicochemical properties or sensory acceptance. Static in vitro digestion demonstrated enhanced intestinal bioaccessibility of vitamin D_3_, while vitamin B_12_ remained stable throughout digestion, indicating that the fortified yogurt could provide physiologically relevant amounts of both micronutrients. Amiri et al. [71] investigated the release behavior of charantin from bitter melon extract-loaded nanoliposomes incorporated into yogurt under simulated gastrointestinal conditions. Charantin release was limited during the gastric phase (20.2–30.4% within 2 h) and increased during the intestinal phase, reaching 35.3–54.3% within the first 30 min, 63.1–82.9% after 3 h, and 71.2–95.0% at the end of intestinal digestion, depending on lecithin and extract concentrations. In addition, Robles-García et al. [72] reported that anthocyanin-loaded nanoliposomes improved color stability, reduced syneresis, enhanced water-holding capacity, and provided sustained release during 21 days of refrigerated storage, while Karakuş et al. [73] showed that peptide-loaded nanoliposomes reduced syneresis, improved textural properties, modulated post-acidification, and enabled controlled gastrointestinal release over 14 days of storage.

In bakery systems, evidence on liposomal delivery remains limited. Sheikhzadeh et al. [74] reported that omega-3 fatty acids encapsulated in soy lecithin nanoliposomes showed high encapsulation efficiency (95.51%) and a mean particle size of 119 nm when incorporated into wheat–corn bread. Compared to free omega-3 addition, liposomal incorporation improved thermal stability during baking and resulted in higher moisture content and lower hardness during storage, indicating delayed staling.

In fresh pepper fruits, *p*-coumaric-acid-loaded liposomes applied directly to the fruit surface showed a markedly enhanced antifungal effect against *Botrytis cinerea* compared to the free compound, reducing lesion area by 55.2%, whereas free *p*-coumaric acid reduced lesion area by 36.6%, both relative to the control fruit, 6 days after incubation. This improved disease control was accompanied by reduced postharvest deterioration, contributing to an extended shelf life during storage [75].

The application of liposomes in food systems has demonstrated improvements in both oxidative and microbiological stability, thereby extending the shelf-life of various food products [42,76]. This enhancement is attributed to their ability to incorporate antioxidant and antimicrobial compounds of different natures, effectively contributing to the preservation of product quality [41,42]. Recently, the combination of liposomes with emerging technologies, including their incorporation into edible coatings and films, the use of techniques such as electrospinning, and their integration with cyclodextrins, has been explored [41,77]. These synergies have enabled the design of more effective food matrices, enhancing both the protection and functionality of the encapsulated bioactive compounds [78,79].

## 3. Combination of Liposomes with Other Technologies

### 3.1. Liposomes Combined with Edible Coatings

Edible coatings are a thin layer of biopolymeric material that covers food to protect it from physical, chemical, and biological contamination. Edible coatings can slow food spoilage and decomposition by acting as selective barriers to the migration of gases, moisture, and solutes during processing, handling, and storage [14]. It has been noted that these coatings can be made using proteins, lipids, and polysaccharides [80]. Combining edible coatings with liposomes represents a novel approach aimed at enhancing their protective and functional performance. The direct incorporation of liposomes into solid foods such as fruits and meat products presents technological challenges related to interactions with complex food matrices, physicochemical stability, and the degradation of encapsulated bioactive compounds [14,81]. Therefore, combining them with edible coatings offers the advantage of placing bioactive agents on the surface of solid products and modulating their release [14]. This approach has allowed for different improvements over traditional coatings, maintaining the functionality of encapsulated bioactive compounds such as antioxidant capacity and antimicrobial activity, thus extending the shelf-life of the foods on which the edible coating is applied [81].

#### Application of Liposomes Combined with Edible Coatings in Different Food Matrices

The application of edible coatings in combination with liposomes has proven to be a promising strategy for improving the preservation and quality of various food groups. The most commonly used technique for the preparation of liposomes intended for use in food products has been the thin-film hydration method (Table 3), which involves the evaporation of the organic solvent, such as chloroform, ethanol, methanol, or dichloromethane, from the lipid component of the liposome to produce a thin-film, followed by the addition of the aqueous phase. In some cases, an additional step is required to provide sufficient energy to form liposomes, for example, by applying ultrasound [82].

Chitosan has been the main material used in the development of edible coatings. It has been reported that the incorporation of liposomes into chitosan films is effective due to the high viscosity of the chitosan solution and the complex formed by the negative (−) charge of the liposomes and the positive (+) charge of the amino groups of chitosan [83]. In the case of meat products, they are highly susceptible to microbial spoilage, color degradation, and lipid oxidation [14]. The studies presented in Table 3 employ essential oils and polyphenol extracts, taking advantage of their antioxidant and antimicrobial properties. These edible coatings delay lipid oxidation, as evidenced by lower peroxide values (PVs) and thiobarbituric acid reactive substances (TBARs) [3,4,14,83], while also reducing the proliferation of spoilage and pathogenic microorganisms such as *Escherichia coli*, *Staphylococcus aureus*, and *Vibrio parahaemolyticus* [4,84]. Furthermore, the sensory characteristics of meat products were not negatively affected by the application of liposome-enriched edible coatings [3,14,83,84].

Fruits are highly susceptible to physical damage and fungal spoilage, resulting in changes to their physicochemical properties and a shortened shelf-life [85]. In the studies shown in Table 3, bioactive compounds (D-limonene and rutin) and aqueous extracts of *Melissa officinalis* were used in the development of edible coatings to prolong the postharvest quality of strawberries, blackberries, almond and tomatoes [85,86,87,88]. This was achieved due to the antimicrobial properties of these compounds, which also contributed to reducing weight loss and ethylene production while improving visual appearance during storage [88]. The evaluated parameters, including weight loss, respiration rate, total soluble solids, titratable acidity, and pH, were not negatively affected during fruit storage with the edible coating.

Edible coatings containing liposomes have been studied in soft white cheese and Karish cheese. Karish cheese is a soft cheese commonly consumed in Egypt, which can be stored at 4 °C for only two weeks or less due to its high moisture content and low salt concentration [89]. Because of the characteristics of both cheeses, they are highly susceptible to microbiological contamination. The incorporation of an edible coating with liposomes containing thyme essential oil effectively reduced the total count of aerobic bacteria, total psychrotrophic bacteria, yeasts, and molds, thereby extending the shelf-life of Karish cheese [89]. Moreover, the addition of nisin to the edible coating was found to reduce the counts of Bacillus cereus and Enterococcus faecium in soft white cheese [90].

The studies shown in Table 3 indicate that combining liposomes with edible coatings is a promising strategy for food preservation, particularly in perishable products such as meat, fish, fruits, and fresh cheeses. Chitosan- and polysaccharide-based coatings incorporating liposomes loaded with essential oils or phenolic compounds enhance antimicrobial activity, delay lipid oxidation, and extend shelf-life under refrigerated conditions. However, most studies are limited to short-term, laboratory-scale applications, with scarce information on long-term stability, industrial scalability, and comprehensive sensory or consumer acceptance evaluations, highlighting the need for further research.

### 3.2. Liposomes Combined with Edible Films

Edible films are thin layers of food-grade biopolymers that provide an environmentally friendly option for food packaging. This type of packaging offers not only a mechanical barrier but also a chemical one [91]. The composition of the edible film or its combination with other technologies can enhance these chemical properties. Certain biopolymers, such as chitosan, possess antibacterial and antifungal properties, providing a greater advantage than plastic packaging by extending the shelf-life of food [92]. However, while the films themselves are biodegradable, their production often involves solvent casting, which requires energy for drying and solvent recovery. Therefore, life cycle assessments are important to validate the net environmental benefit [93].

The combination of edible films with liposomes allows for the incorporation of various bioactive compounds from essential oils, phenols, and other sources. These bioactive compounds can be introduced through liposome technology, which protects them from light, temperature, and pH [94]. The inclusion of these bioactives adds further value to the packaging, providing antioxidant and antimicrobial activity [95], thereby transforming the packaging from passive to active.

Regarding the preparation of edible films containing liposomes, both components are typically prepared separately. The most common method for liposome formation is the thin-film technique. Edible films, on the other hand, are made from biopolymers such as chitosan, gelatin, carrageenan, and starch, each offering distinct properties and advantages [96]. Chitosan is one of the most widely used biopolymers in this field, as it is a cationic polysaccharide that imparts antimicrobial and antifungal properties to the biofilm [97]. Conversely, gelatin contributes to the formation of flexible films [98]; carrageenan can form films with superior mechanical properties, such as high tensile strength and Young’s modulus [99]; and starch is used for its abundance and biodegradability [100]. These characteristics allow films to be tailored to different needs in food preservation and enhancement.

#### Application of Liposomes Combined with Edible Films in Different Food Matrices

The use of edible films combined with liposomes in the meat industry is extensive, ranging from beef to shrimp. The polysaccharides employed vary, indicating that there are no specific polymers required for meat products. Chitosan remains one of the most widely used polymers in the development of edible films due to its high biocompatibility and antibacterial and antifungal activity [81]. Regarding liposome suspensions, the thin-film hydration method is the most frequently used, primarily because of its simplicity [33].

In the studies reviewed, many researchers introduced modifications to the thin-film method, such as incorporating a sonication step. Sonication helps reduce liposome size and achieve a more homogeneous sample [25].

Research on this type of packaging shows that the chemical properties of meat are not negatively affected, as seen in Table 4. In meat products, *Escherichia coli* O157:H7 is one of the most dangerous pathogens for public health. Cui et al. [101] developed a chitosan-based edible film (400 mg/mL) combined with liposomes loaded with specific phages targeting *E. coli* O157:H7. After one day of storage at room temperature, samples treated with the film showed a significant 2.79 log CFU/mL reduction in the pathogen compared to untreated samples, demonstrating the effectiveness of this strategy as an antimicrobial control method [101].

The use of essential garlic oil encapsulated in liposomes for the development of edible films has also been reported, showing effectiveness in preserving meat products. In refrigerated sausages, these films delayed oxidation and the growth of psychrotrophic bacteria, coliforms, and *Staphylococcus aureus* for up to 50 days [102]. Similar results were observed in refrigerated chicken breast filets stored for 14 days [103]. Moreover, the incorporation of essential oil did not alter the sensory properties of the products.

Amjadi et al. [104] developed edible films composed of ZnO, chitosan nanofibers, and gelatin, incorporating betanin-loaded liposomes to wrap beef. This combination of technologies enhanced the antioxidant and antimicrobial properties of betanin, proving effective during 16 days of storage at 4 °C. Treated samples exhibited less formation of secondary oxidation compounds and reduced proliferation of *S. aureus* and *E. coli* compared to samples coated with films containing nonencapsulated betanin or without betanin, highlighting the potential of this technology [104].

Similarly, Luo et al. [105] developed a liposome/chitosan-based coating film for litchi preservation by incorporating thyme essential oil and copper nanoparticles into liposomes. The combined system (liposome–essential oil–CuNPs) exhibited stronger antimicrobial and preservative effects than coatings containing liposomes alone or liposomes loaded only with essential oil, significantly inhibiting the growth of *Colletotrichum* and *Rhizopus*, as well as *E. coli* and *S. aureus*. This synergistic effect reduced browning and decay and extended the storage of litchi fruit up to 9 days compared to the uncoated control [105].

The application of this combination in dairy products is not as extensive as in meat products. However, Niaz et al. [106] developed edible films with nisin-loaded liposomes using ultrasonication, successfully extending the shelf-life of cheese. These films effectively inhibited the growth of pathogens such as *Listeria monocytogenes*, *Staphylococcus aureus*, *Pseudomonas aeruginosa*, and *Escherichia coli* during 18 days of refrigerated storage, demonstrating the potential of this technology to improve the microbiological quality of dairy products [106].

The studies shown in Table 4 demonstrate that the combination of liposomes with edible films is a promising strategy for developing active food packaging, particularly for meat products, where strong antimicrobial and antioxidant effects are consistently reported. Chitosan- and protein-based films incorporating liposomes loaded with essential oils, bacteriophages, or bioactive pigments effectively extend shelf-life without adversely affecting product quality. However, most studies are conducted under short-term and laboratory-scale conditions, and information on long-term stability, large-scale processing, and performance under real packaging and distribution scenarios is still limited. Moreover, sensory evaluation and consumer-related data are often limited or qualitative, and only a small number of studies have addressed the application of these systems in other food matrices, such as fruits and cheeses.

### 3.3. Liposomes Combined with Electrospinning

Electrospinning is a versatile, simple, cost-effective and efficient technique used to produce nano and microfibres with a high surface-to-volume ratio from food-compatible polymers, either natural or synthetic [107]. The application of this technology in the food area can be oriented to the development of packaging materials, food production, food analysis, and other fields [108]. The obtention of the nanofibers is based on the breaking of surface tension of a polymeric solution subjected to the application of a high electric potential. This phenomenon provokes the ejection of a polymeric jet with a conical structure known as Taylor’s cone, being the solvent evaporated to reach a solid, thin mesh material (mat) composed of nanofibers [109]. Furthermore, several factors such as: electrospinning parameters, polymeric solution properties, and environmental factors condition the dimensions and morphology of the nanofibers [110].

The high versatility of electrospinning to process natural, synthetic and thermolabile polymers allows it to electrospun them alone or blends [107,109]. Specifically, the processing of polymer blends allow to obtain nanofibers with improved properties. Among the most frequently used synthetic polymers are polyvinyl alcohol (PVA), polyethylene oxide (PEO), and ε-polylysine, all of which exhibit high biocompatibility [111]. For natural and thermolabile polymers, gelatin and chitosan are the most studied. Most nanofibers are prepared from combinations of these polymers and benefits as biocompatibility, low cost, excellent physico-chemical properties, among others, are expressed in the nanomaterials [112]. Furthermore, in alignment with green chemistry principles, green electrospinning has emerged as a key trend that utilizes solvents like acetic acid, water, and glycerol to fabricate nanofibers. While this approach significantly reduces environmental and health concerns associated with toxic organic solvents, nanofibers derived from water-based polymer solutions often exhibit inferior mechanical properties, compromising their applicability, particularly in aqueous environments [113].

#### Application of Liposomes Combined with Electrospinning in Different Food Matrices

At the moment, the combination of liposomes with electrospinning has been scarcely studied and applied in the food area. This fact is mainly associated with compatibility of matrixes and compounds. To achieve this combination, the liposome formulation must be mixed with the polymer solution before to be electrospun. This is the most common method for loading liposomes onto nanofibers, primarily due to its simplicity, as it requires neither additional equipment nor prolonged processing time. The thin-film hydration method is one of the most frequently used techniques for liposome formation associated with this combination, not necessarily due to compatibility between the two methods, but rather due to its reproducibility and ease of implementation. The combination of liposomes with electrospinning offers several advantages in the food packaging industry, providing antimicrobial, antioxidant, antifungal, and other functional properties [114].

Liposomes combined with electrospinning for meat products have been shown not to alter the physicochemical properties of the meat and, at the same time, to extend its shelf-life [115,116] (Table 5). Regarding encapsulated bioactive compounds, only essential oils have been used to extend the shelf-life of meat products by combining electrospinning with liposomes. The properties of these oils contribute antioxidant and antibacterial activity to biopolymer-based nanofibers. Only one study that evaluated in plant products has been found to explore this combination [117]. This study investigated the effect of nanofibrous films with liposomes on fungi. The authors describe that the use of baicalin, a flavone encapsulated in liposomes and incorporated into nanofibers made with polyvinyl alcohol, reduced weight loss by 50% compared to uncoated mushrooms, thus improving preservation and delaying bacterial growth [117].

The studies shown in Table 5 indicate that the combination of liposomes with electrospun nanofibers is a promising but still emerging strategy in the food sector, mainly explored in meat products, while applications in plant-based matrices remain limited. Systems based on biocompatible polymers such as polyvinyl alcohol, chitosan, and polyethylene oxide incorporating liposome-encapsulated essential oils show enhanced antimicrobial activity and shelf-life extension without adversely affecting food quality. However, the number of available studies remains very limited, with most applications restricted to essential oils and short-term storage evaluations. In addition, challenges related to liposome–polymer compatibility, long-term stability of encapsulated compounds, scalability of electrospinning processes, and the application of these systems to a wider range of food matrices represent important gaps that should be addressed in future research.

### 3.4. Liposomes Combined with Cyclodextrin

Cyclodextrins (CDs) are non-toxic cyclic oligosaccharides composed of a series of α-D-glucopyranose subunits linked by α-1,4-glycosidic bonds. Geometrically, they are characterized by a truncated conical structure with a hydrophilic exterior. Their hollow conical cavity, approximately 7.9 Å in depth, is suitable for the inclusion of hydrophobic guest molecules of appropriate size [118]. The three native cyclodextrins, alpha-CD (α-CD), beta-CD (β-CD), and gamma-CD (γ-CD), contain six, seven, and eight D-glucose subunits, respectively. They are water-stable and exhibit high stability under alkaline conditions. Essential oils have been successfully encapsulated using this system to improve their solubility in aqueous media, enhance chemical stability, reduce volatility, prevent degradation and light-induced losses during processing or storage, and allow for controlled release [119]. Among natural cyclodextrins, β-cyclodextrin is one of the most widely used, as its cavity size is suitable for encapsulating most volatile organic compounds such as essential oils [120]. Inclusion complexes of cyclodextrin/liposomes have been described, aiming to form an aqueous core encapsulating the CD inclusion complex loaded with an active compound, surrounded by one or more phospholipid bilayers [114].

**Table 5:** Applications of liposomes combined with electrospinning and cyclodextrin in different food matrices.

Food	Nanofibers Materials/Cyclodextrin	Active Principle	Liposome Lipid	Liposome Preparation Method	Main Achievement	Reference
**Meat products**						
**Liposomes combined with electrospinning (nanofibers)**						
Chicken	Chitosan and polyethylene oxide	Tea tree oil	Lecithin and cholesterol	Thin-film hydration with some modification	Nanofiber membranes were able to effectively preserve the quality of chicken meat after 4 days of storage.	[115]
Pork	Polyethylene oxide and soy lecithin	Basil essential oil	Soybean lecithin, cholesterol and stearylamine	Thin-film dispersion	This formulation’s materials are safer and more biodegradable and biocompatible than traditional food packaging materials, aligning with current trends in food packaging.	[18]
Shrimp	Polyvinyl alcohol (PVA)	Cinnamon essential oil (CEO)	Lecithin	Thin-film hydration	The PVA nanofiber containing CEO showed stronger antibacterial activity against *S. aureus* and *E. coli* over 12 days, extending shrimp’s shelf-life more effectively than the sample without nanofibers.	[116]
**Liposomes combined with cyclodextrin**						
Pork meat batters	β-cyclodextrin	Nutmeg essential oil	Soy lecithin and cholesterol; cold nitrogen plasma	Thin-film dispersion	This study concludes that their approach is a hopeful solution for maintaining the quality and shelf life of meat batters.	[121]
**Plant products**						
**Liposomes combined with electrospinning (nanofibers)**						
Mushroom	Polyvinyl alcohol and chitosan	Baicalin	Phosphatidylcholine	Reverse evaporation	Nanofibrous films inhibited weight loss, browning, rancidity, and bacterial growth on the mushrooms while maintaining their nutrients.	[117]
**Liposomes combined with cyclodextrin**						
Peach	β-cyclodextrin	Tea tree oil	Soy lecithin and cholesterol	Thin-film dispersion	The coating delayed peach senescence and deterioration. It also inhibited the development of brown rot caused by *M. fructicola*.	[122]

#### Application of Liposomes Combined with Cyclodextrin in Different Food Matrices

To date, only two studies have reported the use of liposome–cyclodextrin combinations with applications in food systems (Table 5). Techniques such as lyophilization and spray drying have been employed to form the liposome–cyclodextrin inclusion complex. Zhu et al. [121] encapsulated nutmeg essential oil in liposomes and subsequently formed an inclusion complex with β-cyclodextrin through lyophilization. This complex was applied to pork meat batters, resulting in a reduction in microbial growth, lipid oxidation, and protein oxidation and degradation during 4 days of storage at 4 °C, compared to pork meat batters without the liposome–β-cyclodextrin inclusion complex. The authors reported an even greater reduction when nitrogen cold plasma technology was applied to the inclusion complex [121].

Additionally, Xu et al. [122] encapsulated tea tree oil in liposomes and then mixed it with a 10% (*w*/*v*) 2-hydroxypropyl-β-cyclodextrin solution (1:4, v:v), stirring for 20 min at 1000 rpm. Finally, the mixture was spray-dried and applied to peach samples. After inoculation with *Monilinia fructicola* L. and 4 days of storage at 20 °C, disease incidence reached 100% in both the inclusion complex and control groups, indicating that infection occurred in all fruits. However, the lesion diameter in the inclusion complex group (23 mm) was significantly smaller than that in the control group (45.80 mm), demonstrating a clear reduction in disease severity.

The studies presented in Table 5 show that the combination of liposomes and cyclodextrins is a promising but still very limited strategy for food preservation. The most promising systems are based on β-cyclodextrin inclusion complexes containing essential oils, further encapsulated within liposomes, as they demonstrate enhanced antimicrobial and antioxidant effectiveness in both meat-based and plant-based matrices. However, the extremely small number of available studies highlights significant gaps in current research, including limited food matrix diversity, scarce information on long-term stability, scalability of drying processes, and the lack of comprehensive sensory and consumer acceptance evaluations. Further studies are therefore required to validate the practical applicability of liposome–cyclodextrin systems in real food preservation scenarios.

### 3.5. Discussion and Future Perspectives

Despite the versatility of liposomal systems demonstrated across food applications, several challenges still limit their industrial implementation [123]. Although their structural tunability through phospholipid composition, lamellarity, and vesicle size has been extensively reported [19,124], key issues related to physicochemical stability, scalability of production methods, cost-effectiveness, and regulatory aspects remain to be addressed before liposomes can be broadly implemented at the industrial scale.

One of the primary limitations relates to the physicochemical stability of liposomes under real food-processing and storage conditions. Factors such as pH, ionic strength, temperature, and the presence of enzymes or surfactants can induce vesicle aggregation, leakage, or membrane destabilization [125,126]. Although the incorporation of modulators such as cholesterol, bile salts, and chitosan has shown potential to enhance rigidity and stability, the development of standardized strategies for stabilizing liposomes within complex food matrices remains an open research need [37,127]. Furthermore, there is limited knowledge regarding the long-term behavior of liposomes in foods with dynamic environments, particularly those undergoing fermentation, thermal treatments, or freeze–thaw cycles.

The valorization of food and agro-industrial by-products as sources of phospholipids and bioactive compounds represents a major opportunity for resource-efficient liposome production. The successful extraction and use of lipids from fish skins, shrimp shells, oilseed residues, and other waste streams support the integration of liposomal technology within the framework of a circular economy [58,128].

Applications in food preservation, particularly in marine and dairy products, have shown convincing improvements in oxidative stability, microbial control, and shelf-life extension when liposomes are loaded with essential oils, oleoresins, polyphenols, or pigments [25,129]. However, most studies remain limited to laboratory scale and often involve immersion, injection, or direct coating techniques that may not be easily scalable or economically viable [130]. A key future direction is therefore the adaptation of liposome incorporation methods to continuous industrial processes, including dipping tanks, spraying systems, and integration with automated packaging operations.

The synergistic combination of liposomes with emerging technologies such as edible coatings, edible films, electrospinning, and cyclodextrin complexes has demonstrated enhanced protection and controlled release of encapsulated compounds. These hybrid systems provide a dual or even triple barrier effect by integrating chemical, physical, and functional protection mechanisms [114,131]. Nevertheless, the interactions, particularly electrostatic interactions, diffusion behaviors, liposome compatibility with the biopolymers, and the reorganization of biopolymer networks are still poorly understood. Advanced characterization tools such as Cryogenic Transmission Electron Microscopy (Cryo-TEM), molecular dynamic simulations, and real-time spectroscopy could substantially improve the understanding of how liposomes behave when embedded in biopolymeric matrices [132,133,134].

Edible coatings and edible films share similar biopolymeric matrices (polysaccharides, proteins, and lipids) but differ in structure and application [135]. Edible coatings are thin layers applied directly onto the food surface, typically by dipping or spraying, forming a primary barrier and being especially suitable for meat, fish, poultry, and fruits and vegetables, where deterioration is mainly associated with oxygen transfer, water vapor permeability, and the growth of spoilage microorganisms at the food–environment interface [80,136]. In contrast, edible films are preformed structures obtained by casting or extrusion and function as packaging materials that wrap the product [137]. When bioactive compounds are directly incorporated into either coatings or films, limitations such as off-flavors, early loss of functionality, and uncontrolled migration away from the food surface may compromise their effectiveness [138]. Therefore, the use of edible coatings or films as encapsulating matrices can help overcome these limitations, and the selection between them depends on the intended application and delivery requirements.

When selecting between electrospinning and conventional edible films, one of the criteria to consider is the release profile of the active compounds. Göksen et al. [139] showed that essential-oil-loaded electrospun zein films, characterized by a porous nanofibrous structure with a high surface-to-volume ratio, provided a more sustained antimicrobial effect on cheese slices, reaching approximately 2 log reductions in *Listeria monocytogenes* and *Staphylococcus aureus* after 28 days at 4 °C, compared with cast zein films, which exhibited a stronger initial effect that decreased during storage. In this context, liposomal encapsulation can be combined with either films or electrospun structures to further modulate release kinetics and improve the stability of sensitive bioactives, depending on the intended application.

Cyclodextrins are primarily selected, compared with the other techniques discussed in this review, when molecular complexation of bioactive compounds is required rather than incorporation into a continuous surface layer [140]. This strategy has been reported for tea tree oil, where cyclodextrins enhance the stability of volatile compounds [122]. In the same study, liposomes were combined with HP-β-CD to obtain spray-dried solid liposomes, improving handling and stability while overcoming the physical instability of liquid liposome systems.

Finally, novel food technologies intended for commercial application must adhere to stringent and evolving safety, labeling, and nutritional regulations established by authorities like the FDA and EFSA, notably under frameworks such as the EU Novel Foods Regulation (Regulation (EU) 2015/2283). This process currently represents a primary translational bottleneck for liposome-based innovations, particularly regarding gastrointestinal fate and toxicity [141]. This regulatory challenge is intensified when liposomes are integrated with emerging technologies—such as edible films or electrospinning—that alter their physical properties, as specific compliance triggers depend heavily on the intended use. Consequently, pathways diverge based on whether the liposomes are ingested systems—potentially falling under “Novel Food” frameworks where the EU’s cross-cutting definition of “nanomaterial” [142] and EFSA’s 2021 guidance necessitate a non-binary risk assessment strategy—or used as food contact materials. For the latter, regulations like the EU Plastics Regulation explicitly require that substances in nanoform be authorized and listed with specific specifications [143]. Furthermore, “food-grade” status is not automatically interchangeable; while lecithin is GRAS in the U.S. [144], purity and process-related impurities can materially alter safety expectations. Ultimately, market deployment faces consumer-facing hurdles like mandatory EU nano-labeling, yet it is imperative that these safety standards do not become overly stringent regulations that inhibit research and development initiatives in the public and private sectors. To ensure future progress, a responsive regulatory policy is required—one that upholds high quality and safety without impeding the implementation of new technologies or stifling the creativity and innovation essential for the field.

In this context, future research should adopt a more translational and forward-looking approach to enable the industrial implementation of liposome-based systems in food applications. Key research priorities include:

- **Standardization of by-products-derived liposomes:** Development of reproducible protocols for lipid extraction and liposome formulation from agro-industrial by-products.
- **Scaling electrospinning for food packaging:** Advancement of scalable and economically viable electrospinning processes using food-grade polymers compatible with industrial packaging systems.
- **Long-term stability in real food matrices:** Evaluation of the physicochemical, microbiological, and functional stability of liposome-based systems under realistic processing and storage conditions.
- **Regulatory and safety aspects:** Assessment of toxicological safety, migration behavior, sensory impact, and regulatory compliance of systems based on the synergistic integration of liposomes.

## 4. Conclusions

The combination of liposomes with complementary technologies such as edible coatings and films, electrospinning, and cyclodextrin inclusion complexes represents a cutting-edge strategy for the development of sustainable and functional food systems. These hybrid approaches not only extend the shelf-life of diverse food matrices, including meat, dairy, fruits, and vegetables, but also enhance their microbiological safety and oxidative stability through the incorporation of bioactive compounds with antioxidant and antimicrobial properties. The synergistic effect of combining liposomes with biopolymeric matrices or nanostructured systems enables the controlled protection and release of sensitive compounds, improving their stability against environmental stressors such as light, oxygen, and temperature. Moreover, these technologies support the transition toward biodegradable and environmentally friendly packaging materials, offering a promising alternative to conventional plastics while maintaining product quality and sensory attributes.

From a technological readiness perspective, however, most of these approaches are still at an early stage of development and have been validated mainly at laboratory scale. Despite the promising laboratory-scale results, several challenges remain before their full industrial implementation can be achieved. Future research should focus on optimizing encapsulation efficiency; improving the physicochemical stability of the hybrid systems under real processing conditions; and developing scalable, cost-effective production methods. Interdisciplinary approaches integrating food engineering, materials science, and nanotechnology will be essential to bridge the gap between experimental findings and industrial applications, consolidating these technologies as viable tools for a more sustainable and resilient food industry.

## Figures and Tables

**Table 2:** Liposomes and their application in the food industry.

Food	Active Principle	Liposome Lipid	Liposome Preparation Method	Main Achievement	Reference
Dried oysters	Rosemary oleoresin (RO)	Lecithin and cholesterol	Thin-film evaporation followed by sonication	RO liposomes have better antioxidant stability during storage and thermal processing properties, which can help prolong food shelf life.	[2]
White cheese	Spirulina extract	Lecithin and cholesterol	Thin-film hydration followed by sonication	Spirulina liposomes inhibit the growth of *Staphylococcus aureus* and *Listeria monocytogenes.* The chemical properties of the cheese were not affected.	[66]
Tilapia slices	Betel leaf ethanolic extracts	Soybean phosphatidylcholine and cholesterol	Thin-film hydration	Treatment could be used to prevent bacterial growth and extend the shelf-life of tilapia slices.	[67]
Sheep cheese	*Thymus capitatus* L. essential oil	Soy lecithin	Thin-film dispersion followed by sonication	Treatment enhanced antioxidant properties.	[68]
Ricotta cheese	Saffron extract	Soy lecithin	Hydration–homogenization followed by ultrasonication	Liposomal encapsulation enabled efficient saffron extract delivery; nanoliposomes showed high physical stability, and increased hardness and chewiness were observed at a saffron extract concentration of 0.125% compared to the control cheese.	[69]
Yogurt	Vitamin D_3_ and Vitamin B_12_	Purified soy lecithins (Phospholipon 90G and Lipoid S45)	Hydration–ultrasonication followed by lyophilization	Lyophilized liposomes were incorporated into yogurt without negatively affecting physicochemical stability or sensory acceptance; in vitro digestion showed enhanced vitamin D_3_ bioaccessibility.	[70]
Yogurt	Bitter melon extract	Soybean lecithin	Hydration–homogenization followed by sonication	Extract-loaded nanoliposomes reduced syneresis, increased viscosity, improved sensory acceptance compared to free extract, and enabled controlled charantin release under simulated gastrointestinal conditions.	[71]
Yogurt	Anthocyanins	Non-hydrogenated soy phosphatidylcholine and cholesterol	Ultrasonic film dispersion	Anthocyanin-loaded nanoliposomes improved color stability, reduced syneresis, enhanced water-holding capacity, and provided sustained release during 21 days of refrigerated storage, while maintaining microbiological safety.	[72]
Yogurt	Bioactive peptides	Purified soybean lecithin	Thin-film hydration followed by ultrasonication and lyophilization	Peptide-loaded nanoliposomes reduced syneresis, improved textural properties, and enabled controlled peptide release during in vitro gastrointestinal digestion, while maintaining microbiological quality during 14 days of refrigerated storage.	[73]
Bread	Omega-3 fatty acids	Soy lecithin	Hydration followed by ultrasonication	Omega-3 nanoliposomes showed high encapsulation efficiency (95.51%), improved thermal stability, increased moisture retention, and reduced hardness of bread, while maintaining sensory acceptability compared to control samples.	[74]
Fresh Pod pepper fruit	*p*-coumaric acid	Soy lecithin and cholesterol	Thin-film hydration method followed by sonication	*p*-coumaric acid-loaded liposomes (83.55 nm) were stable at 4 °C for 25 days, reduced the *Botrytis cinerea* lesion area by 55.2%, and slowed natural decay by 38.7% and weight loss by 37.2%, extending the shelf-life of fresh pod peppers.	[75]

**Table 3:** Application of liposomes combined with edible coatings in different food matrices.

Food	Edible Coating Materials	Active Compounds	Liposome Lipid	Liposome Preparation Method	Main Achievement	Reference
**Meat products**						
Sardine filet	Nanochitosan (chitosan and potassium peroxodisulfate)	*Cumin cyminum* L. essential oil	Soy lecithin and cholesterol	Thin-film hydration and sonication	Coating with liposomes improved the shelf life, microbiological safety, and quality characteristics of sardines during storage at 4 °C for 16 days, according to chemical and microbial analyses.	[83]
Rainbow trout filets	Chitosan (Cs), zein (Zn) and glycerol	*Pulicaria gnaphalodes* (Vent.) Boiss. aqueous extract (PGE)	Soy lecithin and cholesterol	Thin-film hydration and sonication	Coating (Cs-Zn; PGE nanoencapsulated) extended the shelf-life of fish during refrigerated storage by retarding microbial and chemical spoilage. Controlled release of bioactive enhanced antimicrobial and antioxidant properties for 14 days in comparison with the control.	[3]
Salmon filets	Xanthan gum	*Litsea cubeba* essential oil	Soy lecithin and cholesterol	Thin-film dispersion	The 1:3 coating (liposome: xanthan gum, *v/v*) showed the best preservative properties, as it delayed the oxidation of salmon and controlled the growth of *V. parahaemolyticus* at 4 °C.	[4]
Legs of lambs	Chitosan	*Satureja khuzestanica* essential oil	L-a-lecithin and cholesterol	Thin-film hydration and sonication	Controlled release was achieved thanks to the presence of liposomes in the coatings, which decreased microbial counts in lamb meat for 20 days.	[14]
Pork meat	Chitosan and polyethylene	Laurel essential oil and silver nanoparticles	Phosphatidylcholine and cholesterol	Thin-film hydration	The combination of liposomes, laurel essential oil and silver nanoparticles managed to maintain the quality of pork at 4 °C for 15 days, while pure polyethylene films only maintained it for 9 days.	[84]
**Plant products**						
Strawberries	Sodium alginate	D-limonene	Soy-based lecithin and diacetylene	Thin-film dehydration and sonication.	Coatings with limonene liposomes were successful in preserving the quality of strawberries after they were harvested. These coatings were edible and safe for consumption.	[85]
Blackberries	Sodium alginate	D-limonene	10,12-pentacosadiynoic acid and 1,2-dimyristoyl-sn-glycero-3-phosphocholine	Thin-film hydration and sonication.	Edible coatings preserve fruit by controlling gas exchange. Alginate coatings reduce respiration and weight loss, while limonene-liposome coatings reduce mold growth.	[86]
Almond	Hydroxypropyl methylcellulose, cellulose nanofibers, and glycerol	Rutin	Phosphatidylcholine	Heating/homogenization method with lamellarity and size reduction by an ultrasound rod	Liposomes added to almond coating significantly changed density, surface tension and humidity, showing pseudoplastic behavior in temperatures below 25 °C.	[87]
Tomato	Carboxymethylcellulose	*Melissa officinalis* extract	Soy lecithin	Thin-film hydration and sonication	During 10 days of storage at 25 °C and 75% relative humidity, tomatoes with edible coating with liposomes decreased their respiration rate and hydrogen peroxide and malondialdehyde content while preserving their quality compared to tomatoes without the edible coating.	[88]
**Dairy products**						
Karish cheese	Chitosan	Thyme essential oil (TEO)	Lecithin	Thin-film hydration	The shelf-life of Karish cheese can be extended from 2 to 4 weeks with the application of the chitosan-TEO coating	[89]
White soft cheese	Chitosan	Thyme essential oil and nisin	Lecithin	Thin-film hydration	Edible coatings have antibacterial activities against *B. cereus* and *E. faecium.* The shelf life of soft cheese can be extended to 4 weeks with this coating.	[90]

**Table 4:** Application of liposomes combined with edible films in different food matrices.

Food	Edible Coating Materials	Active Principle	Liposome Lipid	Liposome Preparation Method	Main Achievement	Reference
**Meat products**						
Beef	Chitosan	*E. coli* O157:H7 phages	Soy lecithin and cholesterol	Thin-film dispersion	The film achieved the desired antibacterial activity against *E. coli* O157:H7 in the model beef suspension or beef pieces without affecting the quality of beef samples.	[101]
Vacuum-packed sausages	Chitosan or whey protein	Garlic essential oil	Soybean phosphatidylcholine and cholesterol	Thin-film hydration and sonication	Nanoencapsulated herbal EOs are more effective for upgrading packaging films to active packaging composites than free addition, according to this study.	[102]
Chicken breast filet	Chitosan	Garlic essential oil	Phospholipid and cholesterol	Thin-film hydration	The use of a 1% concentration of liposomes in the film extended the shelf-life of chicken filet samples to 2–3 times the usual regulated shelf-life of three days at 4 °C, with inhibitory effects like those of 2%.	[103]
Beef	Gelatin, CH NF, ZnO NPs, glycerol	Betanin	Lecithin	Thin-film hydration	The film, as an active food packaging system, could result in a longer shelf-life for meat products, according to this study.	[104]
**Plant products**						
Litchi fruit	Chitosan (CS)	Thyme essential oil (TEO) and copper nanoparticles (CuNPs)	Lecithin and cholesterol	Thin-film hydration method followed by sonication	Chitosan coating with liposomes containing thyme oil and CuNPs extended litchi’s shelf-life to 9 days, delayed browning and decay, and showed strong antibacterial activity against *E. coli*, *S. aureus*, *Colletotrichum*, and *Rhizopus*. The CS/5% and CS/10% LIP-TEO-CuNPs treatments had the best preservation effect.	[105]
**Dairy products**						
Cheese slices	K-carrageenan, glycerol and nisin	Nisin	Soy lecithin and rhamnolipids	Ultrasonication-assisted homogenization with slight variations	Packaging films with nisin liposomes were more effective at inhibiting bacteria growth in cheese under refrigeration.	[106]

## Data Availability

The original contributions presented in this study are included in the article. Further inquiries can be directed to the corresponding author.
